# Gender-Specific Effect of 5-HT and 5-HIAA on Threshold Level of Behavioral Symptoms and Sex-Bias in Prevalence of Autism Spectrum Disorder

**DOI:** 10.3389/fnins.2019.01375

**Published:** 2020-01-08

**Authors:** Barnali Chakraborti, Deepak Verma, Subhrangshu Guhathakurta, Preeti Jaiswal, Asem Surindro Singh, Swagata Sinha, Saurabh Ghosh, Kanchan Mukhopadhyay, Kochupurackal P. Mohanakumar, Usha Rajamma

**Affiliations:** ^1^Manovikas Biomedical Research and Diagnostic Centre, Manovikas Kendra, Kolkata, India; ^2^Division of Neuroscience, Burnett School of Biomedical Sciences, University of Central Florida, Orlando, FL, United States; ^3^Department of Pathology, University of Mississippi Medical Center, Jackson, MS, United States; ^4^Out Patient’s Department, Manovikas Kendra, Kolkata, India; ^5^Human Genetics Unit, Indian Statistical Institute, Kolkata, India; ^6^Cell Biology & Physiology Division, Indian Institute of Chemical Biology, Kolkata, India; ^7^Inter University Centre for Biomedical Research and Super Speciality Hospital, Mahatma Gandhi University,, Kottayam, India

**Keywords:** gender, behavior, autism spectrum disorder, 5-HT, 5-HIAA

## Abstract

Platelet hyperserotonemia in a subset of Autism Spectrum Disorder (ASD) probands, efficacy of selective serotonin reuptake inhibitors (SSRIs) in reducing behavioral deficits and gender-bias in normal serotonin (5-hydroxy tryptamine or 5-HT) synthesis suggest disruption in stringent regulation of serotonin metabolism in ASD. Therefore, we investigated the changes in 5-HT and 5-hydroxy indole acetic acid (5-HIAA) in ASD probands to assess its effect on the behavior of male and female probands. ASD cases (*n* = 215) were examined using childhood autism rating scale (CARS). Platelet 5-HT (104 cases and 26 controls) and platelet/plasma 5-HIAA (73 cases and 17 controls) were estimated using high performance liquid chromatography coupled with electrochemical detector (HPLC-ECD). In male probands, we observed increase in platelet 5-HT content in association with increase in the score for adaptive responses and increase in platelet 5-HIAA levels with concomitant decline in the score for intellectual response. Age did not influence the neurochemical parameters, but imitation, listening responses and nonverbal communication scores decreased with age. Conversely in female probands, plasma 5-HIAA level significantly attenuated with age, when platelet 5-HT content remained unchanged. Interestingly, platelet/plasma 5-HT and plasma 5-HIAA were higher in female controls. Female probands displayed severe autism-associated behaviors. Overall results indicate gender-bias in 5-HT and 5-HIAA regulation, which probably increases the threshold level of ASD phenotypes in the females, thereby affecting ASD prevalence in a sex-specific manner.

## Introduction

Way back in 1961 Schain and Freedman suggested association of ASD with functionally disrupted serotonin (5-hydroxytryptamine, 5-HT) system, while observing elevated whole blood 5-HT level in affected individuals. Few subsequent studies supported this finding and revealed that the trait is familial (Gabriele et al., 2014). Furthermore, Anderson et al. demonstrated that the increased level of whole blood 5-HT was mainly due to its elevation in the platelets, while maintaining the plasma level in the normal range in ASD probands (Anderson et al., 1987). With similar observations from various groups, platelet hyperserotonemia have become one of the best identified endophenotypes of ASD (Anderson et al., 1990; Hranilovic et al., 2008). Possible contributing factors for platelet hyperserotonemic condition and dysfunction could be increased 5-HT synthesis, decreased degradation of 5-HT and defects in 5-HT signaling mechanisms that involve transporter and receptor functions (Hranilovic et al., 2008; Veenstra-VanderWeele et al., 2012). ASD-associated platelet hyperserotonemia has been a major development that linked ASD with serotonergic dysfunction. Efficacy of selective serotonin reuptake inhibitors (SSRIs) in clinically relieving 5-HT-related behavioral symptoms such as mood, obsession and social relatedness in autistic individuals has given more support to this concept (Buchsbaum et al., 2001; Hollander et al., 2005).

Apart from neurotransmitter function, 5-HT plays a vital role as neurotrophic factor, facilitating neurodevelopment and neurogenesis (Moiseiwitsch and Lauder, 1995). Serotonergic neurons are the earliest formed neurons during mammalian embryogenesis (Shemer et al., 1991) suggesting its neurotrophic function apart from being a neurotransmitter. Developmental defects in metabolic and signaling pathways of the serotonergic system cause disruption of the brain circuitry leading to neurodevelopmental problems, the ultimate manifestation of which usually reflects in behavioral phenotypes. Platelet 5-HT transporter (SERT) has been extensively studied in ASD as it is a key regulator of 5-HT homeostasis in the periphery and the brain (Lesch et al., 1993). Reports on familial nature of the trait (Cook et al., 1993) and animal models of hyperserotonemia mimicking the morphological and behavioral attributes reminiscent of ASD, suggest that systemic elevation of 5-HT levels in ASD has a genetic basis (Whitaker-Azmitia, 2005).

The level of 5-HT metabolite, 5-hydroxyindole acetic acid (5-HIAA) is a possible indicator of 5-HT function. Its level is altered in the cerebrospinal fluid (CSF) as well as in the urine of ASD children, perhaps a compensatory mechanism to maintain the 5-HT level (De Grandis et al., 2010; Adamsen et al., 2014). In general, the 5-HIAA level is higher in infants than in the adults (Hedner et al., 1986) and age dependent alterations impact the complexity behind the disorder. This information is helpful to categorize the subjects based on age for better therapeutic responses. As stated in some studies, 5-HT and 5-HIAA parameters show good correlation with behavioral perturbations (Doudet et al., 1995; Higley et al., 1996) and follow familial mode. These reports strengthen the genetic complexity underlying the disorder (Leboyer et al., 1999). Pharmacological findings indicate that serotonergic system has a physiological role in autism since behavioral responses are mediated through the maintenance of 5-HT and 5-HIAA levels at the synaptic terminals (Duhault and Boulanger, 1977; Raleigh et al., 1984). Accumulating evidences including the gender-specific effect of 5-HT level and function suggest that 5-HT, 5-HIAA, and the severity of ASD symptoms are regulated differentially in males and females. Therefore, we have investigated the correlation between these biochemical parameters and various ASD-specific behavioral alterations, to elucidate the 5-HT mediated complicities in the behavioral phenotype and to analyze the effect of gender on the phenotypic expression.

## Materials and Methods

### Selection of Study Participants

Participants of the study were recruited through the Out Patient’s Department of Manovikas Kendra. ASD diagnosis was done as per the criteria outlined in the Diagnostic and Statistical Manual of Mental Disorders (DSM V) (American Psychiatric Association, 2013). Assessment of ASD cases was performed using Childhood Autism Rating Scale (CARS), a 15-item behavioral rating scale based on observations of various behaviors that categorized ASD patients into mild-moderate and severe cases (Schopler et al., 1980). Scores between 30.0 and 36.5 and between 37.0 and 60.0 are classified as mild to moderate and severe cases, respectively. CARS was assigned for 215 ASD cases (mean age ± SEM: 5.90 ± 0.24) for this study. Study protocol was approved by the Human Ethical Committee of Manovikas Kendra (28042011), which followed the guidelines of Indian Council of Medical Research, Govt. of India. All procedures for the recruitment were performed in accordance with the guidelines and regulations of the committee. Cases and controls were chosen from the same native Bengali speaking population from West Bengal. Detailed family and clinical backgrounds were collected for both the groups. Controls were typically developing children without any known developmental or neurological disorders. After obtaining informed written consent from both cases and controls or their care-givers for voluntary participation and involvement in the study, peripheral blood was collected from the participants. Exclusion criteria for ASD included cases with gross chromosomal abnormalities, and known neurological and/or developmental disorders. Drug naïve candidates were used for unbiased estimation of the neurochemicals (5-HT and 5-HIAA). Age-matched control subjects were selected for the biochemical analyses and for various other correlation studies involving age as a variable.

Total number of control subjects enrolled for this study for platelet 5-HT analysis is 26 (1 to 11.5 years, mean age ± SEM: 5.60 ± 0.61), male controls is 15 (1 to 11.5 years, mean age ± SEM: 5.13 ± 0.82) and female controls is 11 (3 to 11 years, mean age ± SEM: 6.23 ± 0.91). The 5-HT analysis was performed only for 104 ASD cases (2 to 25 years, mean age ± SEM: 5.69 ± 0.37), of which 87 were male cases (2 to 25 years, mean age ± SEM: 5.71 ± 0.42) and 17 were female cases (2 to 15 years, mean age ± SEM: 5.59 ± 0.83). Platelet and plasma 5-HIAA were analyzed for 17 controls (1 to 10 years, mean age ± SEM: 5.47 ± 0.66) and the number of male (1 to 10 years, mean age ± SEM: 5.35 ± 0.93) and female controls was 10 and 7 (3 to 10 years, mean age ± SEM: 5.64 ± 0.99), respectively. ASD cases used for analysis include, 73 cases in total (2 to 25 years, mean age ± SEM: 5.56 ± 0.48), 59 male cases (2 to 25 years, mean age ± SEM: 5.58 ± 0.55) and 14 female cases (2 to 15 years, mean age ± SEM: 5.50 ± 0.92).

### Measurement of Platelet 5-HT, Platelet and Plasma 5-HIAA

Freshly drawn blood was centrifuged at 400 × *g* for 15 min at 4°C to separate RBCs, white blood cells (WBCs) and plasma. The upper plasma and platelet rich plasma (PRP) portions were collected separately. PRP was centrifuged at 1000 × *g* for 5 min at 4°C to collect the platelets as pellet, which was serially washed by resuspending in 1 ml of acid-citrate dextrose, PBS and PBS with glucose. Between each wash, the cells were recovered by spinning at 1000 × *g* for 5 min at 4°C. Finally, it was resuspended in 200 μl PBS containing glucose. From this suspension, 5 μl was diluted 100 times in platelet diluting buffer and 10 μl of this diluted suspension was mixed with an equal volume of trypan blue for counting the platelets in hemocytometer. The remaining platelet suspension was deproteinized by mixing with an equal volume of 0.4 M perchloric acid, sonicated at 50 Hz for 30 s, incubated on ice for 20 min and then centrifuged at 15,000 × *g* for 15 min at 4°C to collect clear supernatant. Similarly, the plasma was also processed by centrifugation at 1000 × *g* for 5 min at 4°C and clear supernatant was collected. It was deproteinized by adding equal volume of 0.4 M perchloric acid, incubated on ice for 20 min and then centrifuged at 15,000 × *g* for 15 min at 4°C to collect clear supernatant. The supernate (10 μl) from both plasma and platelets were injected separately into HPLC-ECD (Bioanalytical Systems, Lafayette, United States) for neurotransmitter analysis (Muralikrishnan and Mohanakumar, 1998; Chakraborti et al., 2016). Here the analytes were separated based on their hydrophobicity using a non-polar stationary phase (C_18_ reverse phase column) and a polar mobile phase. The flow rate was kept at 0.7 ml/min and the electrochemical detection using glassy carbon electrode was performed at 740 mV, for quantification of 5-HT and 5-HIAA with reference to the retention of known standards under same conditions. 5-HT content was determined for 104 cases (mean age ± SEM: 5.69 ± 0.37) and 26 controls (mean age ± SEM: 5.60 ± 0.61) and platelet and plasma 5-HIAA levels were determined for 73 cases (mean age ± SEM: 5.56 ± 0.48) and 17 controls (mean age ± SEM: 5.47 ± 0.66). Comparative analysis of the 5-HT levels between hyper and normoserotonemic ASD groups was done using Mann Whitney U-test.

We have also analyzed the plasma 5-HT level in 93 ASD (age: 5.63 ± 0.37 years; mean ± SEM) and 40 control (age: 5.85 ± 0.54 years) children. The plasma 5-HT level did not show any differences between the two groups. Since we found that it is not altered in the cases and controls, we did not do any further analysis with it.

### Statistical Analysis

Platelet 5-HT, platelet and plasma 5-HIAA levels and CARS score were analyzed using SigmaPlot. The total data set was checked for normality in distribution for each quantifying variables of each group using the Kolmogorov–Smirnov statistic methods. If the normality test fails, then comparison of the differences between two groups was done by Mann-Whitney (M-W) *U* statistics. Tests of correlations between pairs of parameters were done using Spearman’s rank test. All the box plot diagrams and scattered plot diagrams were done using SigmaPlot. Power analysis was performed using online freely available power calculator (https://www.stat.ubc.ca/∼rollin/stats/ssize/). For Mann Whitney test (with α = 0.05, power = 0.80, effect size = 0.8), the total desired sample size was calculated to be 54 using the G^∗^ Power Version 3.1.2 (Faul et al., 2008).

## Results

### Analysis of the CARS Score and Its Comparison Between Male and Female ASD Probands

Distribution of cumulative CARS score and score for each of the 15 behavioral items was estimated for male, female and total ASD probands. The mean value of the scores and their ranges are listed in Supplementary Table S1. Comparison of the mean CARS score values between male and female probands revealed higher score in females than in the males (Table 1). While analyzing the 15 behavioral phenotypes, the mean scores for object use and nonverbal communication were significantly higher in female ASD probands than in the males as given in Table 1.

**TABLE 1:** Mean value of the cumulative CARS score and the score of each behavioral item for ASD Probands.

**ASD probands**	**N**	**Mean score ± SEM**	**Range of**	***t*-test**
			**the score**	***p*-value**
**CARS**				
Total	215	34.92 ± 0.27	29.0–50.0	
Males	180	34.67 ± 0.28	29.0–43.5	**0.050**
Females	35	36.33 ± 0.73	30.0–50.0	
**Object use**				
Total	215	2.53 ± 0.03	1.5–3.5	
Males	180	2.50 ± 0.04	1.5–3.5	**0.029**
Females	35	2.70 ± 0.07	1.5–3.0	
**Nonverbal communication**				
Total	215	2.39 ± 0.03	1.5–4.0	
Males	180	2.37 ± 0.03	1.5–4.0	**0.037**
Females	35	2.54 ± 0.08	2.0–4.0	

According to CARS, the cut off score of the symptoms for autism is 30.0. As mentioned in the Methods, the probands were categorized as mild/moderate or severe based on the CARS score. In the present study total 215 subjects were enrolled which included 180 males and 35 females in a ratio 5:1 (Table 2). Among the 215, 141 were categorized as mild to moderate (66%) and 74 as severe (34%) cases depending on the CARS score. Male ASD probands (180) consisted of 68 and 32% mild to moderate and severe cases respectively. The percentage of mild to moderately and severely affected cases in females were 51 and 49%, respectively. As shown in Table 2 the percentage of severely affected cases were comparatively higher in females than in males.

**TABLE 2:** Percentage distribution of ASD cases based on severity.

**ASD cases**	**Number (n)**	**Percentage of mild to moderate cases (n)**	**Percentage of severe cases (n)**
Total	215	66% (141)	34% (74)
Males	180	68% (123)	32% (57)
Females	35	51% (18)	49% (17)

### Analysis of Platelet and Plasma 5-HT Levels

**FIGURE 1:**
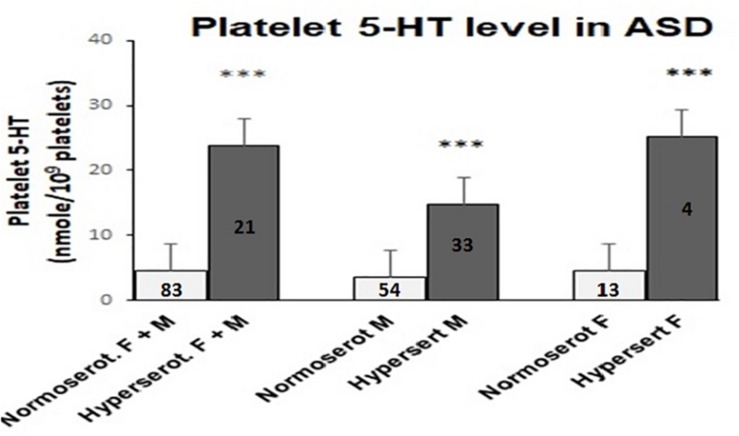
Comparison of platelet 5-HT level in normo- and hyperserotonemic total, male and female ASD probands. The comparative analysis was performed using Mann-Whitney *U* (M-W) test. The number of ASD participants in each study group is depicted inside the bar graphs. ^∗∗∗^ indicates *p*-value to be ≤ 0.005. The power for analysis has been performed assuming equal sample sizes for each ASD group as 52, 44, and 9 for the total (M + F), male (M) and female (F) subjects, respectively. Accordingly, all the three analyses had 100% power.

Analysis of platelet 5-HT level in ASD cases and age-matched controls of same ethnic origin were performed. The cut off value for platelet hyperserotonemia was 14.7 nmol/10^9^ platelets, which was calculated as 95th percentile of the 5-HT value in the controls. In 21 out of 104 ASD cases (20.2%) and 2 out of 26 age-matched controls (7.7%), the observed 5-HT content in the platelets was above this threshold value. Cut off value of hyperserotonemia in male subjects was comparatively less (9.04 nmol/10^9^ platelets). In male participants 33 ASD cases (37.93%) and 1 control child (6.77%) belonged to hyperserotonemic category. On the other hand, females exhibited a higher threshold value of 16.5 nmol/10^9^ platelets for hyperserotonemia and it was present only in 4 ASD cases (23.53%) and 1 control child (9.09%). Comparative analysis of the 5-HT levels using Mann Whitney U-test in hyper and normoserotonemic ASD groups revealed significant increase in platelet 5-HT in hyperserotonemic male, female and control children in comparison to the normoserotonemic groups as shown in Figure 1.

**FIGURE 2:**
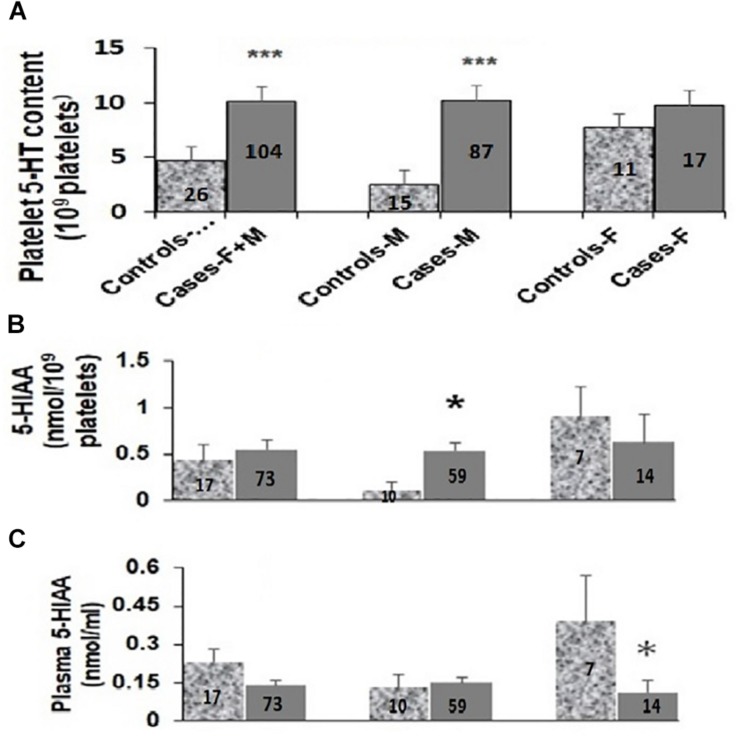
Comparison of platelet 5-HT levels **(A)**, platelet **(B)** and plasma 5-HIAA **(C)** levels between ASD cases and controls. Median values of platelet 5-HT, platelet and plasma 5-HIAA levels in total (F + M), male (M) and female (F) were compared with their respective controls using Mann-Whitney *U* (M-W) test. *Y*-axis of the box-plot diagram shows median 5-HT and 5-HIAA values. Number of subjects for each group is indicated in the bars. ^∗^ indicates *p*-value to be ≤ 0.05, ^∗∗∗^ indicates *p*-value to be ≤ 0.005. Power of this analysis was calculated assuming equal sample size for cases and controls and the power of each analysis is as follows: **(A)** F + M: 51%, M: 84%, F: 69%; **(B)** F + M: 47%, M: 97%, F: 43%; **(C)** F + M: 73%, M: 50%, F: 76%. The respective p-values have been taken as the value for α.

As shown in Figure 2A, the platelet 5-HT levels in ASD cases were 2.5-fold higher than in the controls [controls (N-26): 4.63 ± 1.08 nmol/10^9^ platelets; cases (N-104): 10.08 ± 1.42 nmol/10^9^ platelets; M–W test *U* = 808, *p* = 0.002]. It was further increased to approximately 5-fold in male ASD probands as compared to controls [male controls (N-15): 2.40 ± 0.82 nmol/10^9^ platelets; male ASD cases (N-87): 10.20 ± 1.64 nmol/10^9^ platelets; M–W test *U* = 215, *p* = < 0.001]. On the contrary, in females no significant differences in platelet 5-HT levels were observed between cases and controls as shown in Figure 2A [female controls (N-11): 7.66 ± 1.99 nmol/10^9^ platelets; female ASD cases (N-17): 9.45 ± 2.35 nmol/10^9^ platelets; M–W test *U* = 91, *p* = 0.925]. As mentioned in the Methods section, no differences were observed in the plasma level of 5-HT (ASD cases: 0.09 ± 0.03 nmol/ml plasma; Young controls: 0.08 ± 0.03 nmol/ml plasma; M–W test: *U* = 16.5, *P* = 0.35) between 93 ASD cases and 40 controls.

### Analysis of Plasma and Platelet Levels of 5-HIAA

When plasma 5-HIAA level was compared between total ASD cases and controls, the differences in levels were statistically insignificant [controls (N-17): 0.23 ± 0.05 nmol/ml; cases (N-73): 0.14 ± 0.02 nmol/ml; M–W Test *U* = 474, *p* = 0.126] as shown in Figure 2C. Further analysis based on the sex of the study subjects demonstrated more than 3.5-fold decrease in plasma 5-HIAA levels in the female ASD cases in comparison to age-matched respective controls [cases (N-14): 0.11 ± 0.05 nmol/ml plasma; controls (N-7): 0.39 ± 0.08 nmol/ml plasma, M–W test *U* = 12, *p* = 0.006]. However, no such differences were observed for the male subjects [male controls (N-10): 0.13 ± 0.05 nmol/ml; male cases (N-59): 0.15 ± 0.02 nmol/ml; M–W test *U* = 266.5, *p* = 0.627] as depicted in Figure 2C.

Platelet 5-HIAA level also did not show any statistically significant differences between total ASD cases and controls as shown in Figure 2B [controls (N-17): 0.44 ± 0.17 nmol/10^9^ platelets; cases (N-73): 0.55 ± 0.10 nmol/10^9^ platelets; M–W test *U* = 551, *p* = 0.455]. Interestingly, the platelet 5-HIAA level showed approximately 4.5-fold increase in the male ASD cases as compared to the controls [controls (N-10): 0.12 ± 0.08 nmol/10^9^ platelets; cases (N-59): 0.53 ± 0.10 nmol/10^9^ platelets; M–W test *U* = 180, *p* = 0.040]. However, in the case of female subjects, the difference in the median values were not statistically significant [female controls (N-7): 0.90 ± 0.32 nmol/10^9^ platelets; female cases (N-14): 0.63 ± 0.29 nmol/10^9^ platelets; M–W test *U* = 38, *p* = 0.415] as shown in Figure 2B.

### Gender Specific Changes in 5-HT and 5-HIAA Levels in ASD Cases and Controls

**FIGURE 3:**
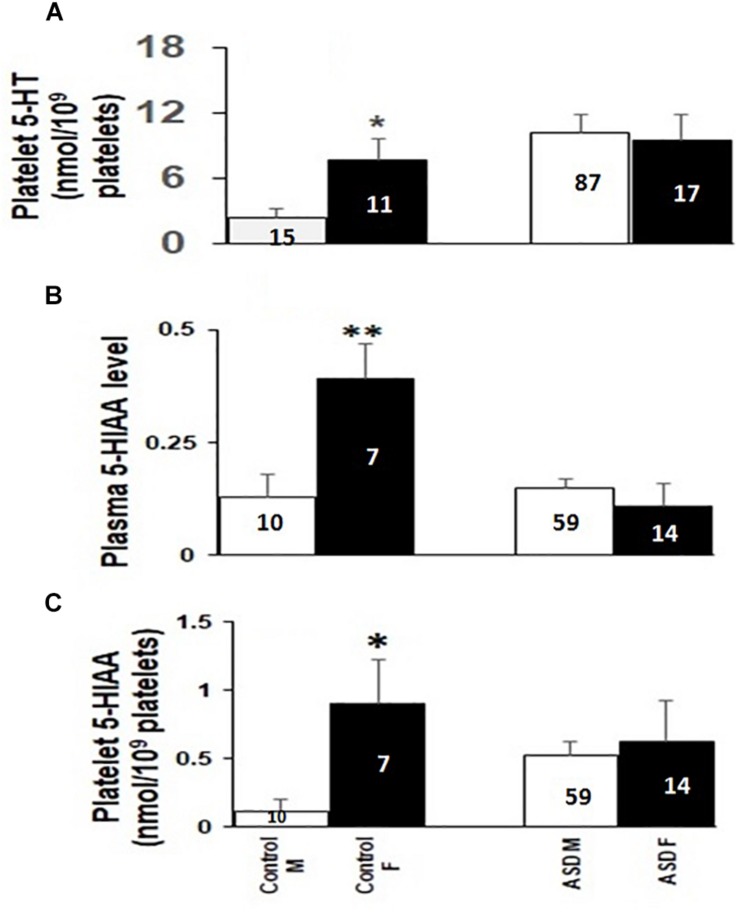
Comparison of platelet 5-HT, plasma and platelet 5-HIAA levels between males and females of ASD cases and controls. The comparative analysis was done using Mann-Whitney *U* (M-W) test. **Panel A** represents comparison of platelet 5-HT, **Panel B** represents comparison of plasma 5-HIAA, and **panel C** represents comparison of platelet 5-HIAA. M and F represent male and female participants, respectively. Left and right bars denote comparison among controls and cases. The numbers inside the bars depicts the number of samples in each group. ^∗^ indicates *p*-value to be ≤ 0.05, ^∗∗^ indicates *p*-value to be ≤ 0.009. Power of the analysis is calculated as: for **(A)** controls: 67%, ASD: 10%; **(B)** Controls: 67%, ASD: 46%; **(C)** Controls: 76%, ASD: 66%

Interestingly, platelet 5-HT, 5-HIAA and plasma 5-HIAA levels in typically developing children showed gender-specific differences among males and females. As shown in Figures 3A–C, all these parameters were higher in the female controls than in male controls. But statistically significant change was not observed in these levels between female and male ASD probands (Figures 3A–C).

### Correlation of CARS Scores of ASD Probands With Platelet 5-HT Levels

Correlation between total CARS score and platelet 5-HT levels in ASD remained insignificant. However, the score for the level and consistency of intellectual response trait indicated a negative correlation with platelet 5-HT levels in total (r_s_: −0.24, *p* = 0.015) and male cases (r_s_: −0.29, *p* = 0.005) as shown in Table 3. None of the other behavioral attributes showed any significant difference.

**TABLE 3:** Correlation analysis of CARS scores of ASD traits with platelet levels of 5-HT, 5-HIAA and age of the probands.

**Behavioral phenotypes**	**ASD subjects**	**Correlation with platelet 5-HT**	**Correlation with platelet 5-HIAA**	**Correlation with age of the proband**
		**r_s_, *p*-value**	**r_s_, *p*-value**	**r_s_, *p*-value**
Relating to People	Total	0.00, 0.997	0.07, 0.536	−0.05, 0.473
	Male	0.02, 0.858	0.07, 0.598	−0.08, 0.354
	Female	−0.04, 0.876	0.00, 1.00	0.09, 0.651
Emotional Response	Total	−0.04, 0.720	0.04, 0.681	−0.08, 0.290
	Male	−0.05, 0.660	0.04, 0.763	−0.05, 0.556
	Female	−0.01, 0.974	−0.02, 0.952	−0.27, 0.175
Imitation	Total	−0.09, 0.324	−0.03, 0.777	−**0.15, 0.044**
	Male	−0.12, 0.282	−0.05, 0.708	−**0.18, 0.028**
	Female	0.06, 0.824	0.04, 0.868	0.04, 0.840
Body Use	Total	−0.07, 0.472	−0.11, 0.358	0.12, 0.108
	Male	−0.09, 0.415	−0.12, 0.374	0.08, 0.337
	Female	−0.00, 0.981	−0.16, 0.573	0.27, 0.169
Object Use	Total	−0.03, 0.749	0.10, 0.381	−0.07, 0.366
	Male	−0.00, 0.991	0.19, 0.141	−0.08, 0.353
	Female	−0.13, 0.618	−0.27, 0.340	−0.06, 0.750
Adaptation to Change	Total	0.07, 0.496	**0.24, 0.040**	0.07, 0.300
	Male	0.11, 0.302	**0.28, 0.033**	0.09, 0.227
	Female	−0.03, 0.906	0.02, 0.952	0.06, 0.752
Listening Response	Total	0.01, 0.935	0.04, 0.706	−**0.15, 0.042**
	Male	0.04, 0.699	0.08, 0.538	−**0.21, 0.009**
	Female	−0.07, 0.773	−0.14, 0.615	0.09, 0.622
Taste, Smell, Touch	Total	−0.05, 0.635	−0.07, 0.573	−0.12, 0.108
	Male	−0.04, 0.704	−0.01, 0.954	−0.12, 0.141
	Female	−0.00, 0.989	−0.38, 0.178	−0.18, 0.359
Visual Response	Total	0.05, 0.590	0.06, 0.613	−0.06, 0.440
	Male	0.15, 0.169	0.02, 0.895	−0.05, 0.523
	Female	−0.24, 0.351	0.23, 0.407	−0.06, 0.752
Fear or Nervousness	Total	0.05, 0.590	0.18, 0.118	−0.02, 0.751
	Male	0.07, 0.515	0.08, 0.540	0.00, 0.988
	Female	0.04, 0.869	**0.58, 0.028**	−0.16, 0.415
Verbal Communication	Total	−0.12, 0.245	0.01, 0.944	−0.04, 0.595
	Male	−0.12, 0.280	−0.01, 0.969	−0.05, 0.528
	Female	−0.03, 0.906	−0.01, 0.964	−0.00, 0.987
Activity Level	Total	−0.05, 0.618	−0.07, 0.575	−0.05, 0.510
	Male	−0.02, 0.868	−0.06, 0.640	−0.05, 0.531
	Female	−0.09, 0.751	−0.08, 0.785	0.04, 0.823
Nonverbal Communication	Total	−0.04, 0.671	0.20, 0.087	−**0.23, 0.002**
	Male	−0.09, 0.390	0.15, 0.251	−**0.22, 0.007**
	Female	0.20, 0.431	0.29, 0.293	−0.21, 0.276
Level and Consistency of Intellectual Response	Total	−**0.24, 0.015**	−0.13, 0.265	0.04, 0.587
	Male	−**0.29, 0.005**	−0.22, 0.092	0.06, 0.438
	Female	0.08, 0.744	0.17, 0.552	−0.17, 0.383
General Impression	Total	−0.18, 0.067	−0.01, 0.927	−0.04, 0.635
	Male	−0.19, 0.069	−0.02, 0.897	−0.04, 0.603
	Female	−0.07, 0.795	−0.15, 0.583	−0.06, 0.750
Overall CARS score	Total	−0.12, 0.239	0.02, 0.862	−0.13, 0.072
	Male	0.10, 0.349	0.01, 0.923	−0.14, 0.077
	Female	−0.11, 0.680	0.05, 0.856	−0.12, 0.542

### Correlation of CARS Scores of ASD Probands With Plasma and Platelet Levels of 5-HIAA

Even though the total CARS score failed to show any correlation with both platelet and plasma 5HIAA, the score for adaptation to change was positively correlated with platelet 5-HT level in total ASD cases (r_s_: 0.24, *p* = 0.040), which was basically contributed by the male probands (r_s_: 0.28, *p* = 0.033). Similar positive correlation was observed in female ASD cases for fear or nervousness (r_s_: 0.58, *p* = 0.028) as shown in Table 3.

### Correlation of Platelet 5-HT, Plasma and Platelet 5-HIAA Levels With Age of the Probands

Platelet 5-HT and platelet 5-HIAA did not show any correlation with age of the probands for both ASD cases and controls. However, a significant negative correlation was observed between plasma 5-HIAA and age of the female ASD probands (r_s_: −0.58, *p* = 0.028) with no effect in any of the controls (r_s_: −0.74, *p* = 0.333).

### Correlation of the CARS Scores With Age of the Probands

Overall CARS score did not show any correlation with age of the probands (Table 3). On the contrary, few specific behavioral attributes such as imitation (r_s_: −0.15, *p* = 0.044), listening response (r_s_: −0.15, *p* = 0.042) and nonverbal communication (r_s_: −0.23, *p* = 0.002) showed significant negative correlation with the age of the probands for total subjects. This effect was mainly contributed by male ASD probands (imitation - r_s_: −0.18, *p* = 0.028, listening response - r_s_: −0.21, *p* = 0.009, nonverbal communication - r_s_: −0.22, *p* = 0.007). However, in the female ASD cases none of these traits showed significant correlation.

### Correlation of Plasma 5-HIAA and Platelet 5-HIAA Levels With Platelet 5-HT Levels

Surprisingly, neither plasma nor platelet 5-HIAA levels showed any significant correlation with platelet 5-HT levels in total, male or female subjects. Absence of correlation was also observed between plasma and platelet 5-HIAA levels for the studied subjects.

## Discussion

Present study highlights the importance of serotonergic system in the gender-specific phenotypic expression, symptom threshold and male prevalence of ASD. The very first observation of elevated levels of the neurotransmitter, 5-HT in the whole blood of ASD children has pioneered the research on neurochemical basis of autism (Schain and Freedman, 1961). Many focused research by independent researchers that followed this, has led to the identification of platelet hyperserotonemia as the first peripheral biomarker of ASD, which is seen only in a subset of affected persons (Anderson et al., 1990). Clinical manifestation of ASD is heterogeneous and it is presumed that alterations in serotonergic system have an impact on behavioral symptoms. Outcome of the present study confirms the earlier findings of platelet hyperserotonemia as an endophenotype of ASD, which we observed only in males. This is accompanied by an increase in platelet 5-HIAA levels in the male probands. On the contrary the 5-HIAA level in the plasma of female cases was low in comparison to the controls, but this trend was not observed in the male ASD participants. Moreover, behavioral symptoms were found to be more severe in the females than males and the distribution of symptom severity based on CARS showed that proportion of mild-moderate to severe cases (2.1:1) was higher in males, whereas it is reduced to 1:1 in ASD females. Of the various behavioral phenotypes mentioned in CARS, the scores for object use and nonverbal communication deficits were higher in females than in male ASDs. When correlating these parameters with various phenotypes, it was observed that the platelet 5-HT content of the male ASD probands was inversely correlated to the score for intellectual ability and consistency. Plasma level of 5-HIAA was directly proportional to the score for adaptation to change in the male ASD probands. Our study indicates that the phenotypic score for imitation, listening response and nonverbal communication decreased with age in male ASD probands. One pertinent observation of this study is the presence of high level of 5-HT and 5-HIAA in the female controls, which implies that gender-bias exists in the distribution of these parameters normally. All these findings for the first time reveal that there is a differential impact of 5-HT and 5-HIAA on ASD phenotypes, which is different in the male and female probands. However, the results should be dealt with caution due to the limited cohort of control subjects when stratified by sex.

Despite high male prevalence of the disorder, most of them exhibited low level of phenotypic expression. On the other hand, proportion of females that displayed severe phenotypes was high. It implies that in the females the threshold for phenotypic expressions is high so that it is diagnosed with the existing tools only when they exhibit higher level of disability. Extreme male brain hypothesis states that during embryonic stage the affected male subjects are probably exposed to high levels of fetal testosterones while in the uterus, which probably explains the increased prevalence of ASD among them (Baron-Cohen, 2002). Another lead in this direction led to the concept of female protective model (Robinson et al., 2013). A previous study suggested that the clinical manifestation of the disorder in females require a higher mutational burden (Jacquemont et al., 2014). From these studies it has been revealed that when ASD phenotype manifests in females it turns out to be more severe. Our present study revealed that females exhibited high CARS scores, which was mainly due to the high scores for object use and nonverbal communication. Happe and his group suggested that females need to have higher degree of behavioral or intellectual disabilities to get diagnosed with the condition (Dworzynski et al., 2012). A later study by Frazier et al. suggested that girls with ASD diagnosis have low IQ and extreme behavioral problems and under such conditions those girls with reduced phenotypic expression are missed out during diagnosis (Frazier et al., 2014). As shown in the Supplementary Table S1, the mean score for level and consistency of intellectual response is comparatively higher in the female probands even though it is not statistically significant, suggesting that females with ASD have relatively low intellectual ability.

Social behaviors are mainly regulated by the neurotransmitter, 5-HT. As it has been observed, platelet 5-HT level is elevated in ASD probands, especially in males. Platelet hyperserotonemia is a consistent finding in a subset of ASD individuals (Anderson et al., 2002; Hranilovic et al., 2008). Our current study also replicated hyperserotonemia in more than 20% of the ASD cases. Differences in platelet 5-HT level between hyperserotonemic and normoserotonemic probands in both male and female probands are also significant. When compared between males and females, 5-HT and 5-HIAA levels showed significantly higher level in female controls than in males. 5-HT levels have been shown to be sexually dimorphic and there exists a sex specific enhancement of platelet 5-HT in male ASD cases (Weiss et al., 2005; Chakraborti et al., 2016). Our present study indicates that 5-HT level of hyperserotonemia is 1.8-fold high in females in comparison to male ASDs. Differential expression of the phenotype in males and females is presumably a functional effect of this altered 5-HT level, acting either through the 5-HT-mediated signal transduction or through its neurotrophic effect. Evidences also exist for the sex-specific effect of SSRI in treating depression (Dalla et al., 2010; Hiroi et al., 2015).

Possible reasons for this sex specific platelet hyperserotonemia in ASD are many amongst which notable ones are: decrease in the activation of the melatonergic pathways arising from an increase in miR-451, miR-375, and miR-7 leading to a decrease in 14-3-3 and AANAT stability in ASD, thereby coordinating increased serotonin with decreased melatonin (Pagan et al., 2017). This altered melatonin hold strong relevance to sex differences in ASD susceptibility as melatonin is a known inhibitor of the estrogen receptor-alpha (Pagan et al., 2014). Second notable reason could be increased oxidative stress and immune-inflammatory activity in ASD, which can drive tryptophan down the kynurenine pathway, linking to data indicating a role for increased quinolinic acid in ASD (Lim et al., 2016). This could contribute to perhaps transient alterations in serotonin e.g., an increased in gut permeability, common in ASD, would drive oxidative stress/cytokines/LPS that may take tryptophan away from serotonin synthesis thus affecting the serotonin level. Thirdly various reports are there which indicates the effect of estrogen in influencing the behavioral phenotype in a sexual dimorphic fashion as estrogen has opposite effects in males and females due to differences in their brain organization (Gillies and McArthur, 2010). There is also burgeoning evidence that serotonergic function is modulated by estrogen which ultimately regulates a variety of behaviors such as mood and cognition (Amin et al., 2005). All these reasons clearly put forth the probable background for the sex specific enhancement of platelet hyperserotonemia and its associated downstream behavioral abnormalities which are observed in ASD.

Even though platelet 5-HT level is increased in the probands, there is lack of correlation with the severity of overall phenotypic expression based on CARS scoring. However, significant negative correlation of platelet 5-HT level was observed with the score of level and consistency of intellectual response in male participants. It suggests that probands with platelet hyperserotonemia has less problem in their intellectual level and cognitive stability (Salgueiro et al., 2012). In many cases the intellectual ability of ASD probands are usually above average and Crespi (2016) in a recent review suggested autism as a disorder of high intelligence. A previous report by Cook et al. (1988) suggested that vocabulary score in normal participants showed negative correlation with whole blood 5-HT (Cook et al., 1988). In a recent paper it has been reviewed that serotonergic system plays a role in cognition, which is modulated by serotonergic receptors (Švob Štrac et al., 2016). Hyperserotonemic condition in platelets and in presynaptic region may be a compensatory mechanism to maintain its level in circulation or in the synapse, where they exert their activity.

Our findings also revealed gender-specific differences as there is 3-fold increase in platelet 5-HT in the females as compared to male controls under normal conditions. The 5-HT level may increase because of a lift in exposure and reuptake of the free circulating 5-HT to the platelet and/or the decreased catabolism of the neurotransmitter in the system. In this study, there is an increased level of its metabolite, 5-HIAA in the platelets of male probands, which indicates abnormalities in 5-HT degradation and clearance of 5-HIAA in the ASD cases. System is fine-tuned to the increased 5-HT level by increasing its degradation. On the other hand, females normally have high basal 5-HT level, which is not different in the affected females also. Females show decreased catabolism of 5-HT as is evident from the low 5-HIAA level in the plasma. However, the 5-HIAA levels are high in females in comparison to male controls. Analysis of the two situations reveals that the increased metabolism of 5-HT in ASD females is to compensate for the elevation of 5-HT level in them. Our previous genetic reports reveal association *MAO* genes with autism, and that also in a gender specific manner (Verma et al., 2014; Chakraborti et al., 2016). We have also shown that 5-HT transporter gene influences ASD (Jaiswal et al., 2015). Therefore, it is likely that genetic influence of serotonergic system genes has a regulatory role on the mechanism and maintenance of serotonergic function and homeostasis, which is differentially modulated in female and male ASD subjects. It is also apparent from the results that the mechanism by which 5-HT regulates the behavioral phenotype is different in boys and girls affected with ASD, possibly in a heritable manner.

Alterations in the levels of 5-HIAA in the blood and urine are not consistent in autism (De Grandis et al., 2010; Adamsen et al., 2014). As platelet serotonergic system in the periphery is supposed to replicate the CNS scenario, the current findings on blood 5-HT and 5-HIAA may reproduce the CNS status of serotonergic profile, however the tryptophan hydroxylases (TPH) in the CNS is different from that of the periphery. Being a neurodevelopmental disorder, ASD-specific neurodevelopmental alterations occur during early fetal stage, during which the TPH in the periphery is only expressed.

Hyperserotonemic condition in CNS results from an increased 5-HT uptake and storage in the presynaptic region, which is likely to reduce the level of synaptic 5-HT for effective neurotransmission. Few neuroimaging studies in ASD demonstrate decrease in 5-HT_2_ receptor binding and reduction in synaptic signal transduction (Murphy et al., 2006; Goldberg et al., 2009). Therefore, possibility of modification of behavioral phenotype through altered serotonergic signaling mechanisms cannot be ruled out. It has been shown that dietary depletion of the 5-HT precursor, tryptophan in drug-free adult ASDs results in worsening of the stereotypic behavior and irritability (McDougle et al., 1996). The findings that SSRIs ameliorate certain ASD behavioral symptoms suggests strong correlation between behavior and 5-HT (Aman et al., 2005; Doyle and McDougle, 2012). Rodent model of knock-in SERT, Gly56Ala recapitulates ASD symptoms like hyperserotonemia, and deficits in social communication as well as repetitive behaviors (Veenstra-VanderWeele et al., 2012). In a recent review by Muller et al. (2016) it has been suggested that serotonergic system is the primary target that can be considered for drug development strategies in ASD, especially in subjects with hyperserotonemia as heritable biomarker.

Concomitant with the increase in 5-HIAA in male ASD probands there is an associated increased score for adaptation to change. It means that 5-HIAA increases the severity of certain parameters of social communication phenotype in these children. On the other hand, female ASD participants showed lower plasma 5-HIAA level, but their platelet level of 5-HIAA remained unaltered, which showed positive correlation with the score for fear and nervousness and results in behavioral inconsistency.

Our findings also indicate lack of correlation between platelet 5-HT levels with either platelet or plasma 5-HIAA levels. The 5-HIAA is excreted through urine and increased level of urinary 5-HIAA has been reported in autism (Hanley et al., 1977; Mulder et al., 2010). Clearance of 5-HIAA through urinary excretion is probably vital to keep the circulatory level in equilibrium and for 5-HT homeostasis. Lack of correlation between serum/platelet 5-HT and 5-HIAA and between platelet and plasma 5-HIAA can be due to the immediate clearance of 5-HIAA via urinary excretion.

Age-dependent change in the expression of the social interaction and communication phenotype is another important finding of this study. Phenotypic expression of the behaviors decreased with increasing age of the probands. The finding is relevant to early intervention programs of ASD for effective management of the deficits in social communication and aberrant behavioral phenotypes. Age-specific effect is restricted to male probands, suggesting gender dependent modulatory effect for age associated alterations in serotonergic system. A previous study demonstrated age dependent decrease in overactivity of 5-HT in healthy children (Dennis et al., 2013).

Current study generates novel and added information to substantiate differential effect of serotonergic system on behavioral expression, which is dependent on the age and sex of a person. The abstract of this study indicates that 5-HT and 5-HIAA are differentially modulated in males and females to have differential effect on age-dependent changes in behavioral expression in males and females affected with ASD. Correlation of male-specific decrease in 5-HT with increased intellectual disability, male-specific low level of 5-HT and 5-HIAA in controls, and presence of apparently mild ASD symptoms in males suggest that males are more vulnerable and need only a low threshold symptom level to express ASD phenotypes. Probably it increases the incidence of ASD in males in comparison to females, where the threshold of symptom level is high. It may be due to high 5-HT and 5-HIAA level, making the prevalence in females low.

This study open avenues for further investigation on 5-HT homeostasis and platelet hyperserotonemia to contribute to variable expression and severity of behavioral symptoms of ASD. Remedial measures to improve behavioral symptoms of ASD are mostly directed to serotonergic system. Serotonergic functions are regulated at the genetic level and ASD-specific behaviors have genetic effect mediated through serotonergic genes (Muller et al., 2016). Our genetic studies on *MAOA* (Verma et al., 2014), *MAOB* (Chakraborti et al., 2016), *VMAT2* (unpublished), and *ENT4* (unpublished) genes supports this concept, and most genes show gender-bias in association. Multiple etiological factors, to name a few the genetic, epigenetic, neurological factors and clinical heterogeneity make ASD highly complex, that needs to be evaluated through an integrated approach. Gender specific phenotypic expression of ASD phenotype and high prevalence of ASD are important areas of research, which need to be evaluated further. As mentioned in the Methods section, for neurochemical analysis we excluded the subjects taking medicines, nutritional supplements and vitamins that could affect levels of 5-HT and 5-HIAA. This is the main reason behind the low sample size. Therefore, the major limitation of our study is the low sample size, especially when the controls are stratified on the basis of gender.

## Data Availability Statement

All datasets generated for this study are included in the article/Supplementary Material.

## Ethics Statement

The studies involving human participants were reviewed and approved by Human Ethical Committee of Manovikas Kendra. Written informed consent to participate in this study was provided by the participants’ legal guardian/next of kin.

## Author Contributions

BC carried out analysis and drafted the manuscript. UR is the Principal Investigator of the study, directed the analysis, prepared and communicated the manuscript. DV, SG, PJ, and AS performed the analysis of neurochemicals. KPM helped in the standardization and subsequent analysis of neurochemicals. SS is the psychiatrist involved in the recruitment of participants and she assessed the severity of symptoms in the ASD children using CARS. SG contributed to statistical analysis. Part of this work was supported by a collaborative grant received by KPM, KM and UR, for which they jointly conceived the idea. This work was also supported by another grant received by UR.

## Conflict of Interest

The authors declare that the research was conducted in the absence of any commercial or financial relationships that could be construed as a potential conflict of interest.
