# Supplementary material for: Gender-Specific Effect of 5-HT and 5-HIAA on Threshold Level of Behavioral Symptoms and Sex-Bias in Prevalence of Autism Spectrum Disorder
Source: Front Neurosci. 2020 Jan 8;13:1375. doi: 10.3389/fnins.2019.01375 (PMC6961535; doi:10.3389/fnins.2019.01375)
Supplement: Supplementary file 1 [file Table_1.DOCX]

**Supplementary Table 1: Distribution of cumulative CARS score and score for each listed behavioral symptom among male, female and total ASD probands and its gender-wise comparison**

| **ASD probands** | **N** | **Mean score ± SEM** | **Range of the score** | **t-test**  ***p*-value** |
| --- | --- | --- | --- | --- |
| **CARS** | | | |  |
| **Total** | 215 | 34.92±0.27 | 29.0-50.0 |  |
| **Males** | 180 | 34.67±0.28 | 29.0-43.5 | **0.050** |
| **Females** | 35 | 36.33±0.73 | 30.0-50.0 |  |
| **Relating to people** | | | |  |
| **Total** | 215 | 2.46±0.03 | 1.5-3.5 |  |
| **Males** | 180 | 2.45±0.03 | 1.5-3.5 | 0.343 |
| **Females** | 35 | 2.53±0.06 | 2.0-3.0 |  |
| **Imitation** | | | |  |
| **Total** | 215 | 2.42±0.03 | 1.0-4.0 |  |
| **Males** | 180 | 2.41±0.04 | 1.0-4.0 | 0.215 |
| **Females** | 35 | 2.51±0.08 | 2.0-4.0 |  |
| **Emotional response** | | | |  |
| **Total** | 215 | 2.26±0.03 | 1.5-4.0 |  |
| **Males** | 180 | 2.24±0.03 | 1.5-3.5 | 0.144 |
| **Females** | 35 | 2.39±0.09 | 1.5-4.0 |  |
| **Body use** | | | |  |
| **Total** | 215 | 2.60±0.04 | 1.0-3.5 |  |
| **Males** | 180 | 2.57±0.04 | 1.0-3.5 | 0.199 |
| **Females** | 35 | 2.73±0.07 | 2.0-3.0 |  |
| **Object use** | | | |  |
| **Total** | 215 | 2.53±0.03 | 1.5-3.5 |  |
| **Males** | 180 | 2.50±0.04 | 1.5-3.5 | **0.029** |
| **Females** | 35 | 2.70±0.07 | 1.5-3.0 |  |
| **Adaptation to change** | | | |  |
| **Total** | 215 | 2.04±0.04 | 1.0-3.0 |  |
| **Males** | 180 | 2.01±0.04 | 1.0-3.0 | 0.241 |
| **Females** | 35 | 2.14±0.10 | 1.0-3.0 |  |
| **Visual response** | | | |  |
| **Total** | 215 | 2.33±0.03 | 1.5-3.5 |  |
| **Males** | 180 | 2.31±0.03 | 1.5-3.5 | 0.067 |
| **Females** | 35 | 2.44±0.08 | 1.5-3.0 |  |
| **Listening response** | | | |  |
| **Total** | 215 | 2.47±0.03 | 1.5-3.5 |  |
| **Males** | 180 | 2.45±0.03 | 1.5-3.5 | 0.133 |
| **Females** | 35 | 2.57±0.07 | 2.0-3.0 |  |
| **Taste smell touch response** | | | |  |
| **Total** | 215 | 1.87±0.03 | 0.5-4.0 |  |
| **Males** | 180 | 1.85±0.04 | 0.5-3.0 | 0.434 |
| **Females** | 35 | 1.95±0.11 | 1.0-4.0 |  |
| **Fear or nervousness** | | | |  |
| **Total** | 215 | 1.64±0.02 | 1.0-3.0 |  |
| **Males** | 180 | 1.62±0.03 | 1.0-2.5 | 0.109 |
| **Females** | 35 | 1.72±0.07 | 1.0-3.0 |  |
| **Verbal communication** | | | |  |
| **Total** | 215 | 2.56±0.03 | 1.5-4.0 |  |
| **Males** | 180 | 2.56±0.03 | 1.5-4.0 | 0.755 |
| **Females** | 35 | 2.60±0.08 | 2.0-4.0 |  |
| **Non verbal communication** | | | |  |
| **Total** | 215 | 2.39±0.03 | 1.5-4.0 |  |
| **Males** | 180 | 2.37±0.03 | 1.5-4.0 | **0.037** |
| **Females** | 35 | 2.54±0.08 | 2.0-4.0 |  |
| **Activity level** | | | |  |
| **Total** | 215 | 2.17±0.04 | 1.0-4.0 |  |
| **Males** | 180 | 2.17±0.05 | 1.0-4.0 | 0.658 |
| **Females** | 35 | 2.13±0.11 | 1.0-4.0 |  |
| **Level and consistency of intellectual response** | | | |  |
| **Total** | 215 | 2.55±0.03 | 1.5-4.0 |  |
| **Males** | 180 | 2.54±0.03 | 1.5-4.0 | 0.310 |
| **Females** | 35 | 2.61±0.07 | 2.0-3.5 |  |
| **General impression** | | | |  |
| **Total** | 215 | 2.64±0.03 | 1.5-3.0 |  |
| **Males** | 180 | 2.62±0.03 | 1.5-3.0 | 0.149 |
| **Females** | 35 | 2.74±0.05 | 2.0-3.0 |  |
